# Editorial: Radiomics in Cardiovascular Imaging

**DOI:** 10.3389/fcvm.2022.876713

**Published:** 2022-04-06

**Authors:** Márton Kolossváry, Damini Dey

**Affiliations:** ^1^Cardiovascular Imaging Research Center, Massachusetts General Hospital, Boston, MA, United States; ^2^Biomedical Imaging Research Institute, Department of Biomedical Sciences, Cedars-Sinai Medical Center, Los Angeles, CA, United States

**Keywords:** radiomics, artificial intelligence, cardiovascular pathology, cardiovascular imaging, cardiac magnetic resonance imaging

Radiomics provides a framework to extract morphological features from medical images, which allows precision phenotyping of pathologies using radiological images (1). Recent investigations in the field of cardiovascular imaging have indicated that these imaging features hold valuable information regarding cardiovascular pathologies above and beyond volumetric analyses (2). In this special Research Topic of Frontiers in Cardiovascular Medicine entitled: “Radiomics in Cardiovascular Imaging,” authors from around the world utilize radiomics to identify new associations between risk factors and cardiac pathologies, differentiate between diseases, and predict patient outcomes using cardiac computed tomography (CCT) and magnetic resonance imaging (CMR).

Age related alterations and sex specific characteristics in cardiac function are well-known. However, it is unclear whether there are morphological changes that can be identified using radiomics. Raisi-Estabragh et al. analyzed tens of thousands of CMR images from healthy participants of the UK Biobank cohort study and found that age, sex, and risk factors had an independent association with radiomic signatures of the ventricle chambers and the texture of the myocardium. In another analysis the authors found that even the foods we eat may have an impact on the morphological characteristics of the myocardium as assessed by CMR radiomics.

Several papers looked into utilizing CMR for the differentiation of cardiovascular pathologies. Rauseo et al. analyzed the data of several hundreds of individuals who suffered from pre-existing ischemic heart disease, ischemic cerebrovascular disease, myocardial infarction, and ischemic stroke. Using 446 radiomic features, they were able to find radiomic signatures specific to each pathology which resulted in significantly better disease discrimination than conventional indices. Izquierdo et al. analyzed over 100 individuals CMRs to differentiate between left ventricular non-compaction, hypertrophic cardiomyopathy, and dilated cardiomyopathy. Using radiomic features of the myocardium (without needing to delineate the trabeculae), the authors were able to differentiate between these pathologies, which may provide a more reproducible methodology to identify these conditions in the future. Wang et al. used radiomics to predict outcomes in individuals with hypertrophic cardiomyopathy. Analyzing 157 radiomic features form 379 consecutive patients, they found that radiomic features provided significant added prognostic value to predict sudden cardiac death, even after adjustments for clinical risk factors and late gadolinium enhancement during the median follow-up of 29 months. Shu et al. found similar results regarding the additive value of radiomics to predict outcomes in individuals with dilated cardiomyopathy with severely reduced ejection fraction. In over 100 patients and a median follow-up time of 505 days, three texture features showed good prognostic value in predicting adverse outcomes, independent of clinical factors and late gadolinium enhancement.

While most articles in this special topic focused on using CMR for precision phenotyping of diseases and prediction of patient outcomes, O'Brien et al. used CCT to identify ischemic scars in the myocardium. Even though CMR is the gold standard for scar detection, the authors show that advanced image analytic techniques are able to extract information from the radiological images and achieve an accuracy of 88%for detecting ischemic scars in the left ventricle on CCT.

Radiomics has the potential to provide precision phenotyping of diseases based on *in-vivo* medical imaging, therefore transforming patient diagnosis, management, and prognostication in the future. The articles published in this Research Topic all push forward the ever-growing field of cardiovascular radiomic research and demonstrate that significantly more information is present in the medical images then that the human eye can apprehend. Radiomics may be able to extract and utilize this information to transform patient care in the near future.

## Author Contributions

All authors listed have made a substantial, direct, and intellectual contribution to the work and approved it for publication.

## Conflict of Interest

The authors declare that the research was conducted in the absence of any commercial or financial relationships that could be construed as a potential conflict of interest.

## Publisher's Note

All claims expressed in this article are solely those of the authors and do not necessarily represent those of their affiliated organizations, or those of the publisher, the editors and the reviewers. Any product that may be evaluated in this article, or claim that may be made by its manufacturer, is not guaranteed or endorsed by the publisher.

## References

[1] Kolossvary M Kellermayer M Merkely B Maurovich-Horvat P. Cardiac computed tomography radiomics: a comprehensive review on radiomic techniques. J Thorac Imaging. (2018) 33:26–34. 10.1097/RTI.0000000000000268 28346329

[2] Kolossvary M Gerstenblith G Bluemke DA Fishman EK Mandler RN Kickler TS. Contribution of risk factors to the development of coronary atherosclerosis as confirmed via coronary CT angiography: a longitudinal radiomics-based study. Radiology. (2021) 299:97–106. 10.1148/radiol.2021203179 33591887 PMC7997618

